# Asymmetry in infants' selective attention to facial features during visual processing of infant-directed speech

**DOI:** 10.3389/fpsyg.2013.00601

**Published:** 2013-09-11

**Authors:** Nicholas A. Smith, Colleen R. Gibilisco, Rachel E. Meisinger, Maren Hankey

**Affiliations:** Perceptual Development Laboratory, Boys Town National Research HospitalOmaha, NE, USA

**Keywords:** infant-directed speech, eye-tracking, face perception, emotion, lateralization, language, speech perception

## Abstract

Two experiments used eye tracking to examine how infant and adult observers distribute their eye gaze on videos of a mother producing infant- and adult-directed speech. Both groups showed greater attention to the eyes than to the nose and mouth, as well as an asymmetrical focus on the talker's right eye for infant-directed speech stimuli. Observers continued to look more at the talker's apparent right eye when the video stimuli were mirror flipped, suggesting that the asymmetry reflects a perceptual processing bias rather than a stimulus artifact, which may be related to cerebral lateralization of emotion processing.

## Introduction

Interaction between caregivers and infants is a complex, bidirectional phenomenon (Cohn and Tronick, 1988; Fogel et al., 1999). This interaction is embodied in the form of a complex combination of dynamic expressions and movements of the face and body, including a characteristic infant-directed (ID) style of speech with distinctive multisensory properties. The goal of this study is to examine how infants process audiovisual samples of ID speech, and examine how this processing might differ from that for adult-directed (AD) speech.

The acoustical properties of ID speech productions have been examined in detail: higher pitch, expanded pitch contours, increased pauses and repetition (Fernald and Simon, 1984; Fernald et al., 1989). Perceptual studies in infants have shown a strong preference for ID speech, (Fernald, 1985; Cooper and Aslin, 1990; Pegg et al., 1992; Werker et al., 1994), and infants' responses have an influence on mothers' speech production during interaction (Smith and Trainor, 2008). Although infants' visual behaviors have often been used as a response measure (as in visual preference procedures) and as a means of understanding infants' multisensory processing of ID speech (Lewkowicz, 1996), much less is known about how infants visually process audiovisual ID speech. This study examines how infants allocate their visual attention on a talker's face when processing ID speech. ID speech is a particularly interesting stimulus because it conveys both phonetic (Kuhl et al., ]bib32; Burnham et al., 2002), prosodic (Fernald and Mazzie, 1991) and emotional information (Trainor et al., 2000; Spence and Moore, 2002), adapted to the infant's developmental state.

Faces are perhaps the most important element of our social environment. Infants show a strong visual preference for faces (Fantz, 1963), which reflects the operation of underlying processes that support the development of face perception and recognition (Morton and Johnson, 1991; Pascalis et al., 1995). The ways in which infants analyze faces vary as a function of a number of factors. Newborns shift from a focus on the peripheral features of the face to exploration of internal features, primarily the eyes, during the first few months of infancy (Maurer and Salapatek, 1976; Haith et al., 1977). A number of studies have shown that over the following months, infants' attention shifts to the mouth (Hunnius and Geuze, 2004). In a variety of speech perception tasks, older infants spent more time fixated on the talker's mouth, which may reflect the increased importance of phonetic information from the talker's mouth at this period of the infant's language development (Lewkowicz and Hansen-Tift, 2012; venbruck, Gervain and Schwarzer, Kubicek et al., 2013; Tenenbaum et al., 2013). In contrast, other work has shown that infants continue to distribute fixations more to talkers' eyes and upper part of the face (Liu et al., 2011). Examinations of mothers' speech productions have shown distinct facial characteristics and exaggerated lip movement in ID speech (Shochi et al., 2009; Green et al., 2010; Shepard et al., 2012).

In most infant eye-tracking studies, the infants are more or less passive observers, making it difficult to ascertain what information-processing goals infants they have, if any, that might guide their selective attention. Studies with adults have explored the role of task on gaze patterns. Eye-tracking studies of audiovisual speech perception have shown that listeners' selective attention to specific regions on the talker's face reflect processing strategies or biases that correspond to attempts to extract information that is relevant to task demands. For example, adult listeners focus on the talker's eyes more when asked to judge emotion or intonation, but direct their gaze to more central or lower regions of the talker's face (i.e., the nose or mouth) when asked to perform difficult speech recognition or segmental judgment tasks (Vatikiotis-Bateson et al., 1998; Lansing and McConkie, 1999; Buchan et al., 2007).

Departures from typical gaze patterns have been observed in people with autism; a hallmark characteristic of which is decreased fixation to the eyes, and increased gaze fixation on the mouth (Klin et al., 2002; Pelphrey et al., 2002). In an effort to explore potential early identifiers of autism, Merin et al. (2007) found that 6-month-old infants at-risk for autism fixated more on their mothers' mouths than did infants in the low-risk control group. However, contrary to expectations, a follow up study of these same infants found that, while not predictive of autism symptoms, infants who fixated more on their mothers' mouths at 6 months showed increased expressive language scores and growth over the next 18 months (Young et al., 2009)—a result that aligns with other studies arguing that increased mouth fixations play a role in speech and language development in infancy (Lewkowicz and Hansen-Tift, 2012; Tenenbaum et al., 2013).

In addition to studies contrasting fixations to the upper and lower faces, other work has explored left/right asymmetries in the processing of faces. Studies using chimeric faces, in which conflicting information is juxtaposed on the left and right side of composite faces, have shown that the right side of faces have a greater influence on observers' classification responses (Levy et al., 1983; Burt and Perrett, 1997). This effect has been called the left visual field (LVF) bias, because the right side of the face appears in the observer's LVF, which projects to processing areas in the right cerebral hemisphere (De Renzi et al., 1994; Haxby et al., 2000; Yovel et al., 2008).

Eye-tracking studies have also demonstrated increased gaze fixation on the right side of faces using photographs (Butler et al., 2005), and the presence of this bias in human infants, rhesus monkeys and domestic dogs (Guo et al., 2009) suggests that this asymmetry reflects a general property of face processing across species. Adults with autism (Dundas et al., 2011) do not show the same LVF bias for facial information as shown by adults in a control group, and a similar lack of LVF bias for still photographs has been shown in infants at risk for autism (Dundas et al., 2012). Using dynamic audiovisual stimuli, other studies have shown similar asymmetries in gaze distributions toward the talker's right eye for talking faces (Everdell et al., 2007), however, many eye-tracking studies of infant speech perception have not contrasted talkers' left and right eyes as separate regions of interest. Given the connection between LVF bias and social cognition, as well as the interactive properties of speech that dynamic audiovisual stimuli approximate (albeit to a limited degree, but more so than still photographs), one might expect to find a LVF bias in infants' visual processing of ID speech, which is arguably as much about the expression and regulation of emotion (Trainor et al., 2000; Spence and Moore, 2002) as it is about phonetic information.

## Experiment 1

### Materials and methods

#### Participants

A total of 39 infants between 5 and 8 months of age were recruited to participate in this study and visited the laboratory. Five infants were unable to complete the eye-tracker calibration procedure, due to fussiness, a lack of interest in the calibration stimuli presented on the screen, or technical difficulties with the eye-tracking system. The 34 remaining infants who did successfully complete calibration, then performed a data validation procedure (described below) to ensure that their recorded eye-tracking data corresponded to known screen locations of validation stimuli. Eighteen infants (age: *M* = 6.04 months, *SD* = 1.04 months, 8 male, 10 female) met the validation criterion, and successfully completed all test trials, and are included in the data analysis below. This experiment was approved by the Institutional Review Board at Boys Town National Research Hospital, and informed parental permission was obtained for all infant participants.

#### Stimuli and procedure

The stimuli consisted of video clips showing a head and shoulders view of a woman engaged in AD and ID speech. In the AD speech condition the talker was recorded for several minutes talking to an adult male, who was seated in line with, but out of view of the camera. In the ID speech condition, the talker was interacting with her own 4-month-old infant, also out of view of the camera. For both ID and AD speech stimuli, the camera was located just above the infant or adult listener's head. This configuration approximated direct eye contact, although imperfectly. The physical presence of the infant is a critical factor in eliciting ID speech (Fernald and Simon, 1984). For each condition, two 30-s-long excerpts were extracted. These were chosen to exclude any vocalizations other than the female talker as well as segments during which the talker's hands may have come into view of the camera.

The videos were presented using custom-written software developed in Max/MSP/Jitter 5, and displayed on a 26-inch LCD monitor (1920 × 1200 resolution, 55 × 35 cm) connected to a Mac Pro computer. The audio portion of the video was delivered through a Roland Edirol FA-101 interface and a Crown D-75A amplifier and ultimately delivered at 55 dBA by a single GSI audiometric speaker mounted slightly above and behind the LCD monitor.

Infants' eye gaze was tracked using a faceLAB 4 eye-tracking system (Seeing Machines Limited, Canberra, ACT, Australia) operating in “Precision” mode, at a rate of 60 samples per second. Real-time data from faceLAB was logged to the stimulus presentation computer, which recorded the gaze screen intersection pixel coordinates with respect to stimulus video time.

Prior to the experiment proper, a short (approximately 1 min) eye-tracking validation procedure was performed in which three looming targets (robot, duck, ball) were presented at three different locations on the screen to ensure that the recorded eye-tracker data corresponded to the target locations. At their maximum expansion these targets subtended approximately 5° in visual angle, and only subjects whose fixations fell within these target regions were included in this analysis. Each subject then performed four trials, two ID speech videos, and two AD speech videos. The stimulus type alternated from trial to trial, with an ID speech stimulus presented first for half of the subjects.

**Figure 1:**
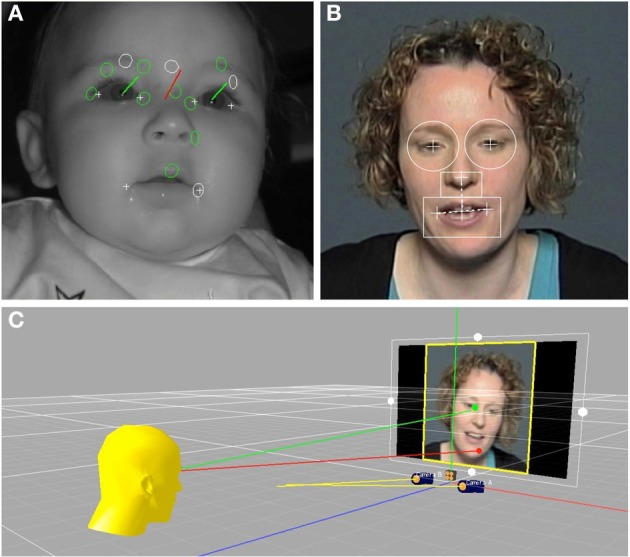
**(A)** Shows an infant during the eye-tracking procedure. The green vectors represent the gaze direction for each eye independently of head orientation, shown in red. **(B)** Shows a frame from one of the video stimuli, with the dynamic regions of interest for the right eye, left eye, nose, and mouth overlain. **(C)** Illustrates the intersection of the infants' eye gaze with locations on the stimulus video.

Because the stimulus talker moved her head in a natural way when speaking, the locations of her facial features in the video varied over time. In order to accurately relate subjects' eye gaze location to these moving facial features, dynamic regions of interests (ROIs) were defined for each video frame of each stimulus using facial feature pixel coordinates, as illustrated in Figure 1. Facial features were found to move over a wider range for the ID than AD speech videos, which corresponds with other work showing exaggerated visual prosodic head movements in ID speech (Smith and Strader, under review). For the left and right eyes, circular regions were defined (5° visual angle in diameter), that were centered on the pupil of each eye. The mouth ROI was defined by a rectangle (10° × 5° visual angle) that was horizontally centered on a bisection point on a line between the left and right corners of the mouth. The top edge of the mouth ROI was vertically aligned with a bisection point between the mouth center point and the tip of the nose. The nose ROI was a rectangle (4° × 2.5°) with a bottom edge that abutted the top of the mouth ROI, which was horizontally centered on the nose tip point.

Prior to analysis, subjects' gaze fixation data and the facial feature location data from the stimulus videos were brought into a common temporal framework. To do this, talker facial feature locations were resampled in MATLAB (The MathWorks, Inc., Natick, MA) from 30 samples per second (the video frame rate) to 60 samples per second (the eye tracker sampling rate) using an interpolation procedure. Given the sampling rate and stimulus duration (60 Hz × 30 s) a total of 1800 eye tracker samples were recorded for each trial.

### Results

**Figure 2:**
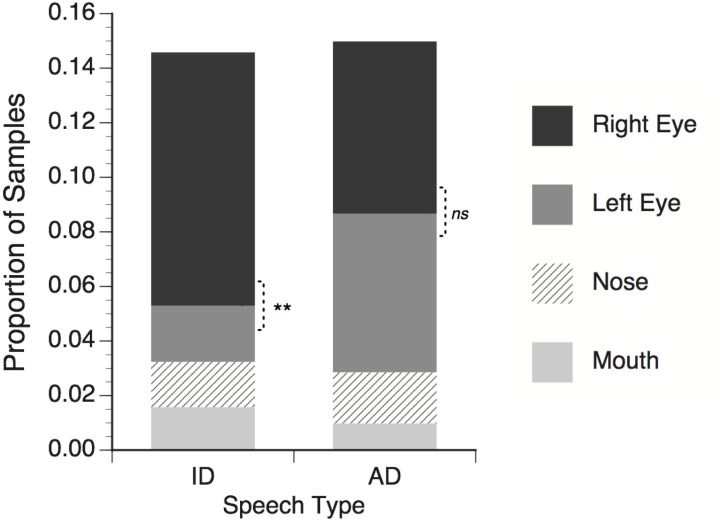
**Proportion of total eye tracker samples in which infants' gaze fell within regions of interest around talker's facial features.** Right eye refers to the talker's right eye, which appeared on the left side of the screen. Significance tests for differences between right and left eye; ^**^p < 0.01, *ns* = non-significant.

On average 67.1% of the eye-tracker samples obtained from infants contained useable eye gaze data. The remaining samples were excluded because of a temporary loss of tracking (e.g., eye blinks, looking away from the eye tracker) or failure to meet the highest gaze quality criterion for at least one eye (level 3 in the faceLAB software). Next, the eye tracking samples were given categorical labels reflecting fixation within the four facial feature ROIs described above: right eye, left eye, nose, or mouth. For each subject and for each trial in the ID and AD speech conditions the proportion of the total number of eye tracker samples falling within each facial feature ROI was calculated, and the average values are shown in Figure 2.

For the both the ID and AD speech conditions, about 15% of the total trial time was spent looking within the four narrowly defined ROIs. Looking proportions can be presented and analyzed in different ways. It is common in other eye-tracking studies to calculate the proportion for each ROI and divide this value by the sum of the proportion values for all ROIs. This essentially normalizes the individual ROI proportions across a range of different total ROI proportions by ignoring all non-ROI looking. While this approach facilitates the construction of index measures (i.e., left vs. right lateralization, or mouth vs. eyes), the proportion of the total stimulus presentation time to which these index measures apply can be obscured, making it difficult to compare the present result with those of previous studies. In the interests of transparency, the present data are reported in terms of proportions of all samples. This captures both the relative differences between each ROI, as well as the relation between all ROI and non-ROI looking.

The mean proportion data for each speech type (ID or AD) were submitted to a two-way repeated measure ANOVA with speech type and facial feature (right eye, left eye, nose, mouth) as within subject factors. No main effect of speech type was found, *F*_(1, 17)_ < 1, *ns*, meaning that proportion of time spent looking at facial features in general did not differ between the ID and AD speech stimuli. A significant main effect of feature was found, *F*_(3, 51)_ = 10.55, *p* < 0.001 η^2^ = 0.383, meaning that across both ID and AD speech stimuli, infants looked at some features (i.e., the eyes) significantly more than others (i.e., nose and mouth). Finally, a significant speech type × feature interaction was found, *F*_(3, 51)_ = 3.97, *p* < 0.001, η^2^ = 0.387, with infants looking significantly more at the talker's right eye than the left in the ID speech condition, *t*_(17)_ = 3.09, *p* = 0.007, but not in the AD speech condition, *t*_(17)_ = 0.30, *p* = 0.77.

The first main finding of this experiment was that infants looked more at the talkers' eyes than the talker's nose and mouth. Although this finding corresponds to other studies showing increased interest in eyes in still images (Maurer and Salapatek, 1976) and moving faces (Haith et al., 1977), it is somewhat in contrast to more recent eye tracking studies that have found a shift in infants' visual attention from the talker's eyes to the mouth starting at 6 months of age, peaking at 10 months, with a return to the talker's eye in adults (Lewkowicz and Hansen-Tift, 2012; Tenenbaum et al., 2013). Similarly, Hunnius and Geuze (2004) found increased fixation to the mouth between 6 and 26 weeks of age, using silent video stimuli. Individual differences in the relative distribution of fixations to the eyes vs. mouth have been found in 6-month-old infants, with subgroups of infants fixating more on the eyes, and others fixating more on the mouth (Merin et al., 2007). The present study tested infants at what would be the beginning of this developmental shift. In contrast to other studies in which more controlled and less interactive speech stimuli were used, the emotional expression may be a particularly salient property of the stimuli used in the present study, thus, drawing infants' gaze to the eyes in an effort to tailor their perceptual processing to emotional information.

The second main finding was a stimulus related lateralization of gaze to the talker's right eye. Although asymmetries have been observed in eye-tracking studies of adults (Everdell et al., 2007), many infant studies have not analyzed or reported eye gaze data with separate ROIs for the talker's left and right eyes (Hunnius and Geuze, 2004; Lewkowicz and Hansen-Tift, 2012; Tenenbaum et al., 2013). The basis for this asymmetry was explored in Experiment 2.

## Experiment 2

Experiment 1 found that infants looked more at the talker's right eye than the left, particularly for the ID speech stimuli. The purpose of Experiment 2 was to compare infants' looking behavior with that of adults, as well as to rule out the possibility that this asymmetry was due to some artifactual property of the talker's right eye that may have attracted the observer's attention, rather than a more general perceptual processing bias. To test this, a group of adult observers performed the same eye-tracking task as the infants, but with two different versions of the stimulus used in Experiment 1. The “original” versions were the same as those used in Experiment 1. The mirror-flipped versions differed only in that the image was horizontally reversed so that the talker's right eye now appeared on the right-hand side of the screen, and appeared as though it were the talker's left eye. If the asymmetry observed in Experiment 1 were due to some property of the talker's right eye, we would expect a reversal of the gaze asymmetry in the mirror-flipped condition. If the asymmetry is due to a processing bias, then observers should continue to look at the apparent right eye in both conditions.

### Materials and methods

#### Participants

Twenty-four adults (5 men, 19 women; mean age = 27.04 years, *SD* = 9.65 years) participated in this experiment. All participants had normal, or corrected-to-normal, vision (on the basis of self report). Three additional adults were recruited but were not included in this sample because of calibration/validation problems with the eye tracker (*n* = 2), or technical difficulties with stimulus presentation (*n* = 1). This experiment was approved by the Institutional Review Board at Boys Town National Research Hospital, and informed consent was obtained from all participants.

#### Stimuli and procedure

The stimuli and procedure were identical to those used in Experiment 1, with the addition of a set of mirror-flipped versions of the videos in which the left and right sides were reversed. Stimuli were presented in random order. Gaze data from the eye tracker was analyzed in terms of ROIs in the same way as in Experiment 1. For mirror-flipped versions of the stimuli, the label “Right eye” refers to what appeared to be the talker's right eye, which was presented on the left side of the screen.

### Results

**Figure 3:**
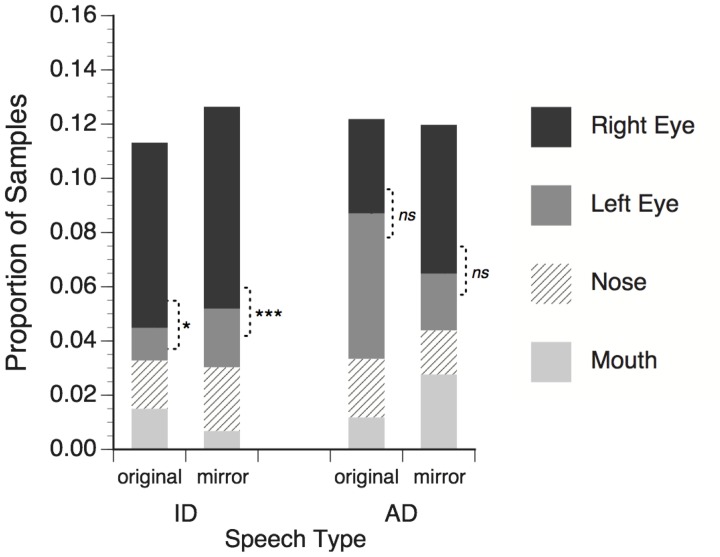
**Proportion of total eye tracker samples in which adults' gaze fell within regions of interest around talker's facial features.** Right eye refers to the talker's right eye, which appeared on the left side of the screen. Significance tests for differences between right and left eye; ^*^p < 0.05; ^***^p < 0.001, *ns* = non-significant.

On average 94.5% of the eye-tracker samples contained useable eye gaze data (defined as quality level 3 for at least one eye in the faceLAB system). Samples that failed to meet this criterion were excluded from the analysis. Eye tracking samples were reduced to categorical labels corresponding to the four facial feature ROIs, and the proportions of samples falling within each ROI were calculated for each trial. The mean proportions, averaged across subjects for each stimulus condition are shown in Figure 3.

Mean proportion data were submitted to a three-way repeated measures ANOVA with speech type (ID or AD speech), facial feature (right eye, left eye, nose, mouth), and mirror reversal (original or flipped) as within-subjects factors. No significant main effects of speech type, *F*_(1, 23)_ < 1, *ns*, or mirror reversal, *F*_(1, 23)_ < 1, *ns*, were found, meaning that observers looked at talker facial features in general similarly for ID and AD stimuli, and for original and mirror-flipped versions of the stimuli. A significant main effect of feature, *F*_(3, 69)_ = 5.73, *p* = 0.001, η^2^ = 0.20, and significant interaction of speech type × feature, *F*_(3, 69)_ = 7.06, *p* < 0.001, η^2^ = 0.235, showed that observers distributed their gaze to some features more than others, and that the pattern of distribution was different for the ID and AD speech stimuli. The speech type × mirror-reversal interaction was not significant, *F*_(1, 23)_ < 1, *ns*.

The speech type × mirror reversal × feature interaction was significant, *F*_(3, 69)_ = 2.91, *p* = 0.041, η^2^ = 0.112. For ID speech stimuli observers looked significantly more to talker's right eye than left for both the original, *t*_(23)_ = 2.43, *p* = 0.023, and mirror-flipped, *t*_(23)_ = 3.83, *p* = 0.001, versions of the stimuli. These effects correspond to the right eye asymmetry of infants in Experiment 1. For AD speech stimuli, no significant difference was observed in the amount of looking to the left and right eye in the original, *t*_(23)_ = 0.96, *p* = 0.348, or mirror-reversed conditions, *t*_(23)_ = 1.68, *p* = 0.106.

The results of Experiment 2 extend the findings of Experiment 1 to adult observers, showing that asymmetrical distribution of eye gaze to the talker's right eye is common to both age groups when processing ID speech. The persistence of this effect for mirror-reversed versions of these stimuli provides evidence that the asymmetry does not reflect a response to an artifactual property of the stimulus, but rather a visual processing bias in observers.

## General discussion

The present study examined where infant and adult observers distribute their eye gaze on the talker's face when watching audiovisual examples of ID and AD speech. Gaze fixations were not uniformly distributed, but rather concentrated on the upper portion of the face. Previous eye-tracking studies provide a varied and changing account of the infants' selective attention to facial features, with some studies demonstrating an increased focus on the eyes (Liu et al., 2011), and others an increased focus on the mouth (Hunnius and Geuze, 2004; Lewkowicz and Hansen-Tift, 2012; Tenenbaum et al., 2013). The first year of infancy is obviously a period of rapid developmental change, and methodological differences between studies make drawing conclusions about infants' visual processing strategies difficult. The present study differs from others, in that the stimuli were recorded in the context of the talker's natural, emotionally expressive interaction with her own infant and an adult listener, rather than simulated ID speech or non-interactive monologue with more controlled speech material. Just as the acoustic properties of mothers' ID speech are influenced by feedback from infants (Fernald and Simon, 1984; Smith and Trainor, 2008), it is also likely that the way in which infants visually process talking faces depends a great deal on the way in which the faces are talking, and the nature of the information communicated. The observed focus on the talker's eyes is consistent with studies of adult observers performing emotional judgment tasks (Buchan et al., 2007).

Although infants consistently looked more at the talker's eyes, an asymmetry was observed in which they looked significantly more at the talker's right eye for ID speech stimuli. This effect was replicated in Experiment 2, in which adult observers showed a similar right-eye distribution for both original and mirror-flipped versions of the stimuli, suggesting that the asymmetry reflects a processing strategy or perceptual bias, rather than the effect of a stimulus artifact. This result opens the question of what property of ID speech is driving the observed gaze asymmetry. Although much of the foundational research on ID speech focused on the acoustical differences between ID and AD, there has been a growing interest in the visible aspects of mother-infant interaction (Brand et al., 2002; O'neill et al., 2005; Green et al., 2010). Recent work has shown increased visual prosodic head movements in ID speech, as well as stronger relations between mothers' voice pitch and head position (Smith and Strader, under review). Could these increased head movements be considered a natural confounding factor? This characterization assumes that ID speech is primarily an acoustical phenomenon, with visible correlates. Although there are practical challenges to creating realistic ID and AD speech stimuli in which head movements are controlled, the growing acknowledgment of ID speech as an integrated multisensory phenomenon suggests that controlling for visual prosody by instructing mother to restrict their head movements will likely produce an artificial and impoverished, rather than a purer, example of this phenomenon. However, the use of animations to independently manipulate the visible head movements associated with speech (e.g., Munhall et al., 2004) may be a promising approach to exploring the factors underlying the visual processing of ID speech.

The increased focus on the talker's right eye likely bears some relation to the LVF bias observed in other studies of face perception. Typically, these studies use chimeric faces, in which the face stimulus contains conflicting information on the left and right sides, such as gender, emotion or attractiveness expressed (Levy et al., 1983; Burt and Perrett, 1997; Alpers, 2008; Parente and Tommasi, 2008). Facial information that appears in the observer's LVF exerts a greater influence on the observer's response than the information on the right visual field (RVF), in perceptual judgment tasks. Because information from the LVF projects to the right hemisphere of the brain, this bias has been interpreted as evidence for the right hemispheric lateralization for face processing (Yovel et al., 2008). Using tachistoscopic presentation of faces to the LVF and RVF, De Schonen and Mathivet (1990) found that infants had enhanced recognition for their mothers' faces when present in the LVF (right hemisphere). Visual input to the right hemisphere during infancy is necessary for the development of face processing expertise (Le Grand et al., 2003).

Eye-tracking studies showing increased focus on the right eye suggest that infants also have LVF bias for photographs (Guo et al., 2009; Dundas et al., 2012). While LVF bias has been shown for dynamic faces in adults (Everdell et al., 2007), the present study provides evidence for a similar asymmetry in infant observers. However, the use of eye tracking introduces some complications related to a LVF bias interpretation, which assumes that observers' eye gaze is centrally fixated on the face. Specifically, if the observer fixates on the talker's right eye then a larger portion of the face shifts to the observer's RVF, which projects to cortical areas of the *left* hemisphere—a somewhat counter-intuitive phenomenon (for discussion of this point see: Butler et al., 2005; Butler and Harvey, 2006; Dundas et al., 2011).

For the sake of argument (and perhaps speculative exploration) if observers' focus on the talker's right eye is indeed about putting the talkers' face in the RVF, where it projects to left cerebral hemisphere, what possible function might this bias serve? One possibility is that it may reflect hemispheric lateralization of emotional processing, described by various theoretical accounts. According to the Approach/Withdrawal hypothesis, the left hemisphere is dominant for the processing of “approach” emotions, such as happiness and anger (Davidson et al., 1990). Similarly, according to the Valence Hypothesis, positive emotions are left hemisphere dominant (Adolphs et al., 2001). Using silent video presentations of happy and sad facial expressions, Davidson and Fox (1982) found that 10-month-old infants showed greater left-hemisphere lateralized activity in frontal regions in response to happy expressions. Although a meta-analysis of neuroimaging studies provides general support for valence-specific lateralization, these effects are complex, with some brain regions showing different lateralization effects than others (Wager et al., 2003). The increased fixation to the talker's right eye, particularly for the ID speech stimuli, may reflect a perceptual strategy for the processing of this kind of emotional stimulus.

Intersecting with ideas about lateralization of emotional face processing are ideas about hemispheric lateralization of speech processing. It has been widely argued that prosodic information is processed by specialized areas in the right hemisphere (Ross, 1981), while linguistic/phonetic information is processed by areas in the left (Zatorre et al., 1992; Vigneau et al., 2006), though this dichotomy is not clear cut (Schirmer and Kotz, 2006; Vigneau et al., 2011). Optical imaging of hemodynamic responses in 3-month-old infants has shown greater right-hemisphere contrasts in response to prosodic differences in sentences, though bilateral effects were also observed (Homae et al., 2006). Similarly, studies using event-related potentials (ERPs) have shown bilateral response to prosodic differences in speech (Shafer et al., 1999). Comparing ERP responses to ID and AD speech, Zangl and Mills (2007) found increased left-hemisphere activation for ID speech in 6-month olds, and increased bilateral responses at 13 months. Although these imaging studies only presented speech in the auditory modality, this result is consistent with an interpretation of our gaze asymmetry finding as reflecting a left-hemisphere involvement in the processing of ID speech. Furthermore, it is possible that a lack of gaze asymmetry may relate to deficits in emotion processing in children—an effect that parallels the lack of a LVF bias in adults with, and infants at-risk for, autism (Dundas et al., 2011, 2012).

The processing of spoken language, presented in the context of multisensory interaction, is a complex task involving the coordination of multiple specialized systems (i.e., for speech, faces, emotion). Understanding the complex interaction between these systems will likely require a coordinated experimental approach in which moment-to-moment relations between visual behaviors and cortical processing can be examined. For example, a concurrent and synchronous combination of eye tracking and ERP would provide a more complete picture of how infants' visual processing biases relate to cortical mechanisms involved in processing this information. Understanding the connection between looking behavior and neural processing will provide a window through which the development emotion and audiovisual speech processing abilities can be examined in typical developing children, as well those with autism.

### Conflict of interest statement

The authors declare that the research was conducted in the absence of any commercial or financial relationships that could be construed as a potential conflict of interest.
